# Osteoclast stimulation factor 1 (*Ostf1*) KNOCKOUT increases trabecular bone mass in mice

**DOI:** 10.1007/s00335-017-9718-3

**Published:** 2017-09-21

**Authors:** Matthieu Vermeren, Rodanthi Lyraki, Sachin Wani, Rannar Airik, Omar Albagha, Richard Mort, Friedhelm Hildebrandt, Toby Hurd

**Affiliations:** 1 Institute for Genetics and Molecular Medicine, University of Edinburgh, Western General Hospital Campus, Crewe Road, Edinburgh, EH4 2XU UK; 2 Rangos Research Center, Children’s Hospital of Pittsburgh, Pittsburgh, PA 15224 USA; 3 Division of Biomedical and Life Sciences, Faculty of Health and Medicine, Lancaster University, Lancaster, LA1 4YG UK; 4 Division of Nephrology, Boston Children’s Hospital, Harvard Medical School, Enders 561, 300 Longwood Avenue, Boston, MA 02115 USA; 5 MRC Centre for Inflammation Research, Queen Medical Research Institute, University of Edinburgh, 47 Little France, Edinburgh, EH16 4TJ UK; 6 MRC Human Genetics Unit, Institute for Genetics and Molecular Medicine, University of Edinburgh, Edinburgh, UK

## Abstract

Osteoclast stimulation factor 1 (OSTF1) is an SH3-domain containing protein that was initially identified as a factor involved in the indirect activation of osteoclasts. It has been linked to spinal muscular atrophy in humans through its interaction with SMN1, and is one of six genes deleted in a human developmental microdeletion syndrome. To investigate the function of OSTF1, we generated an *Ostf1* knockout mouse model, with exons 3 and 4 of *Ostf1* replaced by a LacZ orf. Extensive X-Gal staining was performed to examine the developmental and adult expression pattern, followed by phenotyping. We show widespread expression of the gene in the vasculature of most organs and in a number of cell types in adult and embryonic mouse tissues. Furthermore, whilst SHIRPA testing revealed no behavioural defects, we demonstrate increased trabecular mass in the long bones, confirming a role for OSTF1 in bone development.

**Electronic supplementary material:**

The online version of this article (doi:10.1007/s00335-017-9718-3) contains supplementary material, which is available to authorised users.

## Introduction

Osteoclast stimulation factor 1 (OSTF1) was originally described as SH3P2 in a screen for Src-homology 3 (SH3)-containing proteins by peptide array (Sparks et al. 1996), and independently also discovered in an expression cloning screen (Reddy et al. 1998). OSTF1 indirectly enhances, through the supernatant of transfected 293 cells, osteoclast formation and bone-resorption activity in cell culture assays. Structurally, OSTF1 is a small intracellular protein that contains an SH3 domain closely followed by four ankyrin domains (Tong et al. 2009). Northern blot analysis indicated the presence of a single *OSTF1* transcript in multiple human tissues (Reddy et al. 1998). In a series of cell-based experiments, overexpression of OSTF1 in HeLa cells was found to have a negative impact on cell motility in transwell migration assays (Tanimura et al. 2011). Morphologically, these transfected HeLa cells were found to be more rounded and had a smaller footprint that controls.

OSTF1 has been shown to interact directly with a series of intracellular proteins using several methods, including co-immunoprecipitation, peptide array and yeast two hybrid. Binding partners identified include F-actin (Szymkiewicz et al. 2004), the non-receptor tyrosine kinase c-Src (Reddy et al. 1998; Szymkiewicz et al. 2004) and the E3 ubiquitin-protein ligase Casitas B-lineage lymphoma (Cbl) (Szymkiewicz et al. 2004; Vinayagam et al. 2011). This specific interaction has been shown to be strengthened by the co-localisation of OSTF1 with Cbl in the podosomes of osteoclast-like cells, and has been suggested to be important for their bone-resorption properties (Szymkiewicz et al. 2004). OSTF1 has also been demonstrated to interact with Survival of Motor Neuron 1 and 2 (SMN1 and SMN2 respectively) (Kurihara et al. 2001; Vinayagam et al. 2011), the loss of which leads to spinal muscular atrophy. Both SMN1- and -2 are found in the cytoplasm of neurons from which they translocate to subnuclear bodies called gems, where small nuclear riboproteins are assembled (Massenet et al. 2002; Paushkin et al. 2002). Intriguingly, conditioned media from 293 cells overexpressing SMN has also been shown to drive enhanced formation and hyper-activation of osteoclasts (Kurihara et al. 2001).

Together with three other known genes and two open reading frames of unknown functions, *OSTF1* forms part of a chromosomal region that is deleted in a microdeletion syndrome at 9q21.13 (Baglietto et al. 2014; Boudry-Labis et al. 2013). The deletion leads to mild mental retardation, autism-spectrum disorder, small stature, speech delay and epileptic seizures. OSTF1 is thought to play only a minor role in this syndrome since the mouse knockout of two of the deleted genes, the retinoic acid receptor RAR-related Orphan Receptor B (*RORB*) and the magnesium channel Transient Receptor Potential cation channel, subfamily M, member 6 (*TRPM6*) recapitulate some of the syndrome’s phenotypes (Boudry-Labis et al. 2013). No knockout of the fourth gene in the deletion, Nicotinamide Riboside Kinase 1 (*NMRK1*) has been reported.

Genome wide association studies correlate variation in *OSTF1* to coronary artery diseases, variation in body mass index (Fox et al. 2007), Alzheimer’s disease (Furney et al. 2011), multiple sclerosis (Baranzini et al. 2009) and non-alcoholic fatty liver disease (Chalasani et al. 2010). However, correlation is not causation and the in vivo function of *OSTF1* remains unknown.

Here, we report for the first time a mouse knockout for *Ostf1, Ostf1* ^lacZ/LacZ^, where the coding exons 3 and 4 have been replaced by the LacZ reporter. We observe that the *Ostf1* ^lacZ/LacZ^ mouse suffers from a mild form of osteopetrosis, caused by an increase in trabecular bone. We have taken advantage of the LacZ insertion and extensively analysed the expression pattern of *Ostf1* through X-Gal staining and defined further regions of potential interest for phenotyping.

## Materials and methods

### Accession IDs

Organism (mouse)—Taxon ID 10090.

Gene (*Ostf1*)—Gene ID 20409.

### OSTF1^+/LacZ^ mice

ES cells on the C57BL/6N background for the *Ostf1* ^tm1(NCOM)Cmhd^ mouse, termed *Ostf1* ^LacZ^ line in this paper are available from NorCOMM under the ID NO1551. Chimeric mice were prepared by blastocyst microinjection and bred with C57BL/6J mice to obtain germline transmission. All experimental procedures using animals were approved by The University of Edinburgh’s Animal Welfare and Ethical Review Board and conducted under the Animals (Scientific Procedures) Act 1986. All work was performed under project licence #60/4424. All mice used in this study were euthanized by CO_2_ inhalation.

### Western blotting

**Fig. 1:**
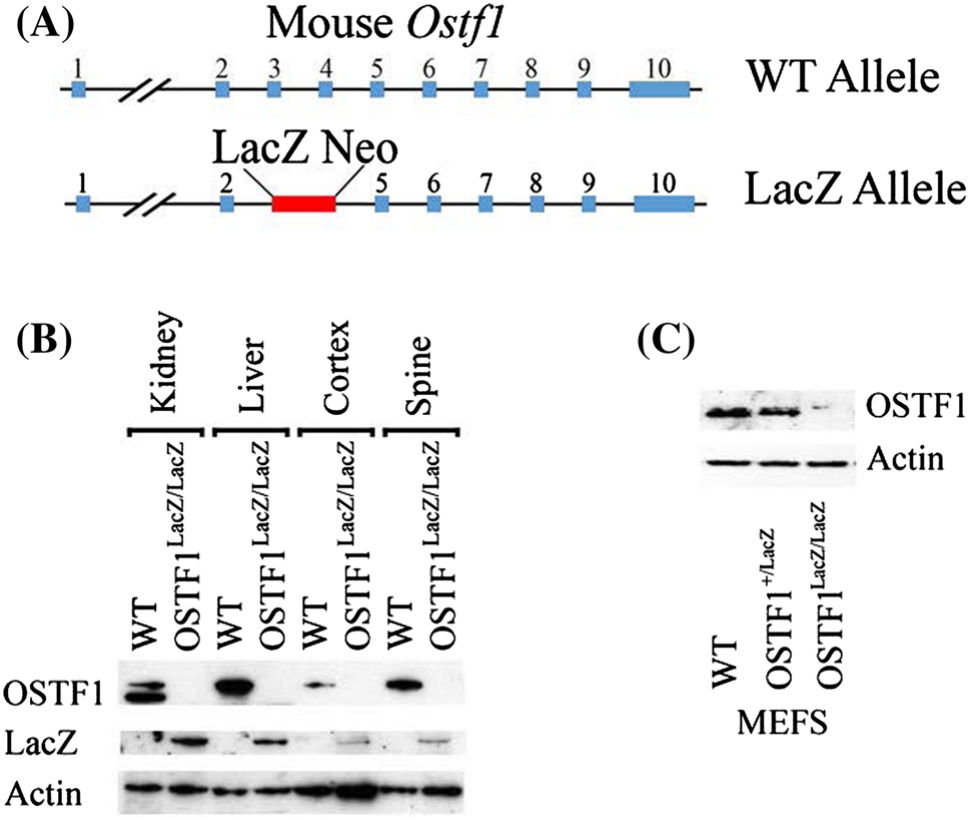
The *Ostf1* ^LacZ/LacZ^ mouse is a knockout with β-galactosidase activity reflecting levels of *Ostf1* expression. **a** Gene knockout strategy: out of 10 exons (blue boxes), exons 3 and 4 are replaced by a LacZ Neo cassette (red). **b** Western blotting to detect endogenous OSTF1 in different tissues. OSTF1 protein expression is observed in wild-type kidney, liver, cortex and spine, but absent in *Ostf1* ^LacZ/LacZ^ tissues. β-galactosidase (LacZ) protein expression is only detected in *Ostf1* ^LacZ/LacZ^ tissues. β-actin was used as a loading control. **c** Expression of OSTF1 in lysates from MEFs derived from wild type (WT), *Ostf1* ^+/*LacZ*^ and *Ostf1* ^LacZ/LacZ^ mice. β-actin was used as a loading control

Western blotting was done using standard procedures. The following primary antibodies were used: rabbit anti-OSTF1 (Bethyl laboratories, A303-004A) at 1:10,000 dilution (Fig. 1a, g, f), rabbit anti-OSTF1 (Atlas HPA020514) at 1:200 dilution (Fig. 1b), rabbit anti-β actin (Neomarkers RB-9421-R1) at 1:100 dilution, rabbit anti-β galactosidase (Invitrogen A-11132) at 1:5000 dilution.

### Genotyping

DNA was extracted from fresh tissue, amplified by PCR and products resolved by gel electrophoresis. Primers used were: Exon 3F AACCACCACTGTATTCTAAGGTAGG, Exon 3R AAACAGTCCAGCACTATGCTAATC, Exon 4F CGGACCCAGATGGTTTCAG, Exon 4R CGTGCGTTTGTCCTGTTG, Exon 9F TTGTTCTCCGGTAGCTTTGC, Exon 9R CTCGCCCTTTGCAGTAAAAC, LacZ F ATCCTCTGCATGGTCAGGTC, LacZ R CGTGGCCTGATTCATTCC. PCR conditions are available upon request.

### LacZ staining

Otherwise mentioned, all products were obtained from Sigma. Tissues were processed for cryostat sectioning followed by X-Gal staining according to standard procedures (Mackenzie et al. 1997). Some sections were counterstained with eosin.

### Generation of mouse embryonic fibroblasts (MEFs)

MEFs were generated from E13 embryos according to standard procedures and cultured in Optimem supplemented with 10% foetal bovine serum, penicillin/streptomycin and 114 µM β-mercaptoethanol. After three passages, MEFS were processed either for X-Gal staining or for protein extraction.

### Imaging

Stained sections were photographed with an upright AX10 microscope (Zeiss) linked to a Retiga 400R camera (QImaging) using a QImagin pluggin for ImageJ/FiJi. Images were processed and assembled using Adobe Photoshop. Some sections were photographed using a Dotslide scanner (Olympus) and images were analysed using the OlyVia software (Olympus).

### CT scanning and bone analysis

Legs of age-matched male WT and *Ostf1* ^*lacZ*/*LacZ*^ mice were isolated and fixed overnight in 4% paraformaldehyde. Muscles were then dissected out and the entire leg was stored in 70% ethanol. Scanning was performed on a Skyscan 1172 Micro-CT scanner (Bruker, Kontich, Belgium) following the protocol described in van’t Hof and Ralston (1997) and van’t Hof (2012). Data was then reconstructed and a slice of bone below the growth plate was selected for quantification as described in Idris et al. (2005).

### Osteoclast differentiation

Legs of age-matched male WT and *Ostf1* ^*lacZ*/*LacZ*^ sibling mice were isolated. Bone marrow from tibia and femurs were expelled from bone shafts using a MEM-containing syringe and 23 gauge needle. Subsequently, bone marrow was triturated to dissociate the cells and then plated in MEM/FCS/Pen-Strep/Glutamine containing macrophage colony stimulating factor (M-CSF, 100 ng/ml, Prospec) to induce macrophage production. After 2 days, M-CSF dependent macrophages were dissociated and 5000 cells were put in each well of a Lab-Tek multichamber. Medium was then changed to MEM/FCS/Pen-Strep/Glutamine containing 25 ng/ml of M-CSF and 100 ng/ml of Receptor activator of nuclear factor kappa-B ligand (RANKL, Prospec) to induce osteoclast differentiation. Cells were left to differentiate for 5 days before processing.

### Tartrate resistant acid phosphatase (TRAP) staining

Fixed osteoclast cultures were TRAP stained according to protocol described in (Idris et al. 2005). Chamber slides containing osteoclasts were photographed with an inverted microscope (Zeiss) and multinucleate TRAP-positive osteoclasts were manually counted.

### Identification of OSTF1-interacting proteins

To identify OSTF1-interacting proteins, HEK293 cells cultured in DMEM/10% FCS/Pen-Strep were transiently transfected in triplicate with either empty vector (pCDNA3.1 V5-His) or V5-OSTF1 plasmids. After 48 h, cells were lysed in IP lysis buffer (50 mM Tris pH 7.5, 1% Triton-X100, 150 mM NaCl), protein content measured and subjected to automated IP on a Kingfisher Duo (Thermo) using anti-V5 magnetic agarose (MBL). Digestion and protein identification was performed by the MRC Institute of Genetics & Molecular Medicine Proteomics Facility.

### Behaviour analysis

Mice tested were age-matched siblings from heterozygotous pairing. Cohorts consisted of six female WT, seven female heterozygotes and six female *Ostf1* ^LacZ/LacZ^, four male WT, three male heterozygotes and eight male *Ostf1* ^LacZ/LacZ^.

1. SHIRPA test was as described in Rogers et al. (1997)
2. Cat Box test: In order to analyse movement and gait, we constructed a 1 m-long, 10 cm wide and 20 cm high Perspex box with a translucent floor located mid-height, 10 cm from the base. A 45° angle mirror was placed under the floor so that the mouse could be observed from the side and from underneath at the same time. Mice were allowed to run the length of the box to a cardboard tube while being filmed using a Canon S500 SLR camera. Movies were converted to the AVI format using Avidemux (http://www.avidemux.org/admWiki/doku.php) before being imported into ImageJ/Fiji (Schneider et al. 2012). A custom macro was then used to record the positions at which each paw made contact with the floor. Step size was measured using Pythagoras’ theorem.
3. Food burrowing test was as described in Yang and Crawley (2009)
4. Preyer’s reflex: We have constructed a click box (https://www.ihr.mrc.ac.uk/projects/clickBox). Preyer’s reflex was tested by clicking once near the tested animal and observing whether its ears twitched.

## Results

### *Ostf1*^*LacZ*/*LacZ*^ is a knockout for *Ostf1*

We took advantage of the availability of an *Ostf1* mouse ES cells, *Ostf1* ^*LacZ*/*LacZ*^, from the North American Conditional Mouse Mutagenesis Project (Fig. 1a). In these mice, exons 3 and 4 that encode most of the *Ostf1* SH3 domain are replaced by a LacZ expression cassette. As a result, the remaining exons are out of frame. As expected, OSTF1 protein was undetectable by Western blotting in *Ostf1* ^lacZ/LacZ^ tissues (Fig. 1b). Instead, LacZ was expressed under the control of *Ostf1* promoter (Fig. 1b). Similarly, reduction and loss of OSTF1 protein was also observed in embryonic fibroblasts derived from *Ostf1* ^+/LacZ^ and *Ostf1* ^lacZ/LacZ^ animals, respectively (Fig. 1c).

Heterozygous mating yielded 151 mice at the ratio of, 40 wild types: 63 heterozygotes: 48 knockouts. Both male and female homozygous *Ostf1* ^lacZ/LacZ^ mice were fertile and without overt deleterious phenotype up until the age of 1 year.

### *Ostf1* expression

In order to define potential function of the *Ostf1*, the spatial and temporal pattern of expression was examined through the use of a combination of X-Gal staining, Western blotting and RT-PCR. X-Gal staining was used extensively since it allows for resolution of expression at the single cell level.

### Central nervous system

**Fig. 2:**
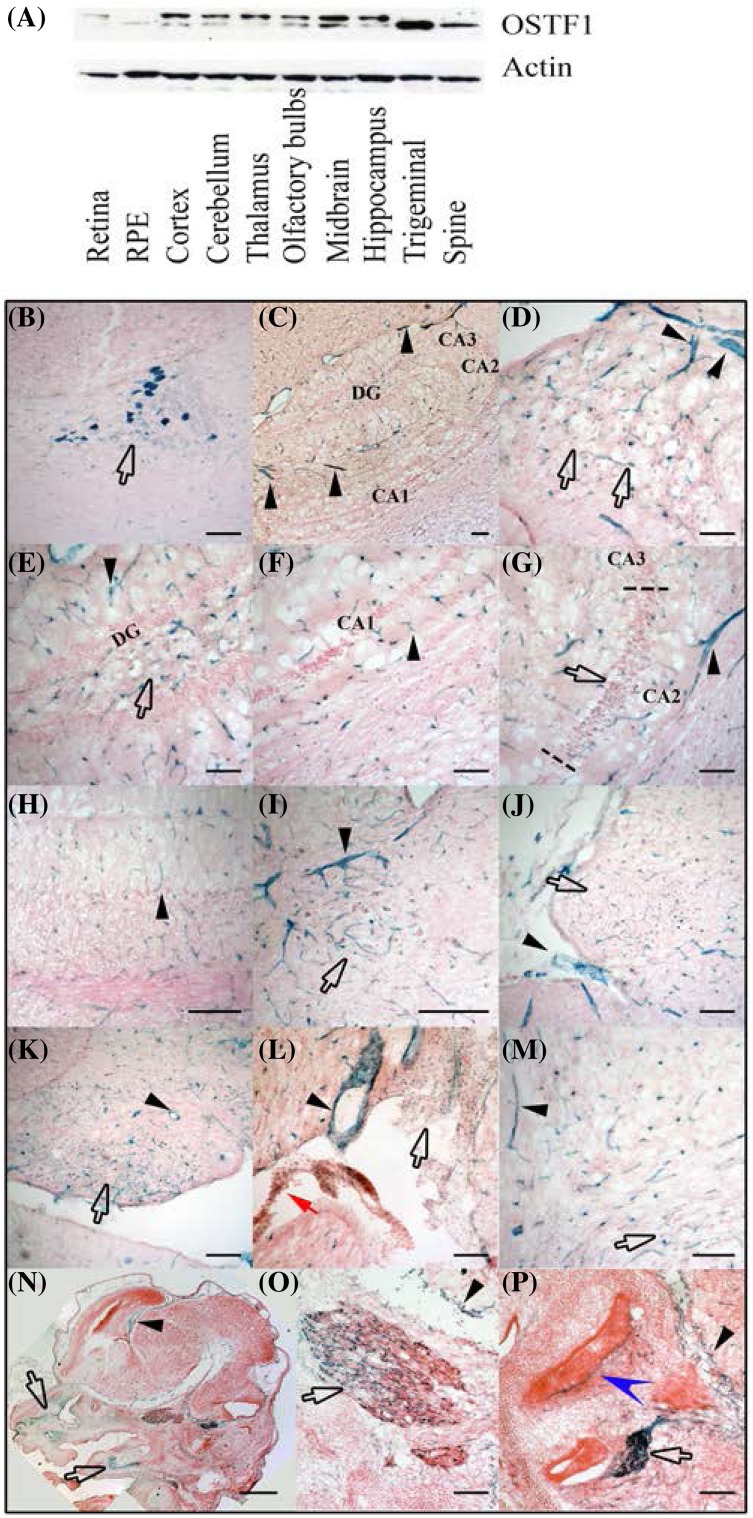
OSTF1 is widely expressed in brain tissue. **a** Tissues belonging to the nervous system were dissected and subjected to western blotting using an anti-OSTF1 antibody with β-actin serving as loading control. *RPE* retinal pigmented epithelium. **b** The parabrachial nucleus expresses the highest level of OSTF1 (arrow). **c**–**g** OSTF1 is found at low level in the hippocampal formation. **d** OSTF1 expression appears as puncta in the subiculum. **e** OSTF1 expression in the polymorph layer of the dentate gyrus (DG). **f, g** The cornus amonis (CA) region 2 (CA2) defined by dashed lines between CA1 and CA3. **h, i** In the cortex of the cerebellum (panel **h**), OSTF1 is only found in blood vessels (arrowhead) but it is consistently present in the deep cerebellar nuclei (panel **i**—arrow). **j, k** Small puncta indicate low levels of expression in the mammillary nucleus (panel **j**—arrow) and the spinal vestibular nucleus (panel **k**—arrow). **k** The ventricular surface (white arrow) and the choroid plexus (red arrow) both express OSTF1. **l, m** Throughout the brain, OSTF1 is highly expressed by the endothelium of the blood vessels (black arrowheads) as exemplified by large and small blood vessels in panel **k, l** and in small branched cells. **n, p** In the E14 embryo head, OSTF1 expression was observed in the blood vessels and the meninges (dark arrowheads). **n** OSTF1 is, however, also present in the cartilage precursor of the skull (arrows). **o** At this stage, the trigeminal neurons express varying levels of OSTF1. **p** In contrast, the spiral ganglia express high levels of OSTF1 and the epithelium of the cochlear epithelium (notched arrowhead). Scale bar = 500 µm, all other panels scale bar = 100 µm

Unlike the recently assumed (Baglietto et al. 2014; Boudry-Labis et al. 2013) and in keeping with the original findings of Reddy et al. (1998), we find that *Ostf1* is widely expressed in the brain, albeit at low levels. Western blotting revealed *Ostf1* expression in virtually all central nervous system regions' analyses, albeit at differing levels (Fig. 2a). Results are summarised in Table 1. The highest level of expression was noted in the neurons of the parabrachial nucleus (Fig. 2b, arrow). Together with axons from the cranial ganglia, the parabrachial nucleus neurons were the only ones to sport neurites that were also clearly labelled. In other regions, LacZ was expressed far more weakly, suggesting that less *Ostf1* is normally expressed. For example, we observed *Ostf1* expression in the hippocampal formation (Fig. 2c): in the subiculum (Fig. 2d, arrows), polymorph region of the dentate gyrus (Fig. 2e, DG, arrow), Cornus Amonis region 2 (CA2) (Fig. 2g, arrow, defined by dashed line) but neither CA1 or CA3 (Fig. 2f, g). In the cerebellum, *Ostf1* was restricted to blood vessels in the cortex (Fig. 2h, black arrow head) and it was observed as small puncta in the neurons of the deep cerebellar nuclei (Fig. 2i, arrow). *Ostf1* was also expressed in the mammilary nucleus and the spinal vestibular nucleus (Fig. 2j, k respectively).

**Table 1:** Table summarising expression pattern of OSTF1 in the adult mouse brain

Main structure	Structure	Level of expression
Everywhere	Blood vessels	3
	Pial surface	2
Cortex	Somatosensory, layer V	1
Hippocampal formation	CA2	1
	Polymorph region of dentate gyrus	1
	Subiculum	1
Thalamus	Submedial nucleus of the thalamus	1
	Reticular nucleus of the thalamus	1
	Mediodorsal nucleus of the thalamus	1
Hypothalamus	Mammillary nucleus	1
Striatum	Amygdalar nuclei	1
Midbrain	Red nucleus	1
	Oculomotor nucleus	1
Cerebellum	Deep cerebellar nuclei	1
Hindbrain	Pontine gray	1
	Pontine reticular nucleus	1
	Superior olivary complex	1
	Tegmental reticular nucleus	1
	Dorsal nucleus raphe	3
	Medial vestibular nucleus	1
	Dorsal cochlear nucleus	1
	Nucleus of the lateral lemniscus	1
	Primary sensory nucleus of the trigeminal	1
	Spinal vestibular nucleus	1
	Paragigantocellular reticular nucleus	2
Spinal cord	Motor neurons	1
	Floor plate	1
	Central canal	1
DRGs	Salt and pepper in all sensory neurons	2–3
Cranial ganglia	Trigeminal	3
	Vestibulocochlear ganglia	3

Throughout the brain, large and also small blood vessels were strongly labelled (dark arrowheads exemplified in Fig. 2l, m). Likewise, the lining of the brain, the ventricles as well as the choroid plexus (Fig. 2l, white and red arrows) were LacZ positive. Finally, everywhere except in the cerebellum, small branched cells, presumably oligodendrocytes and their precursors were observed to express *Ostf1* (Fig. 2m).

**Fig. 3:**
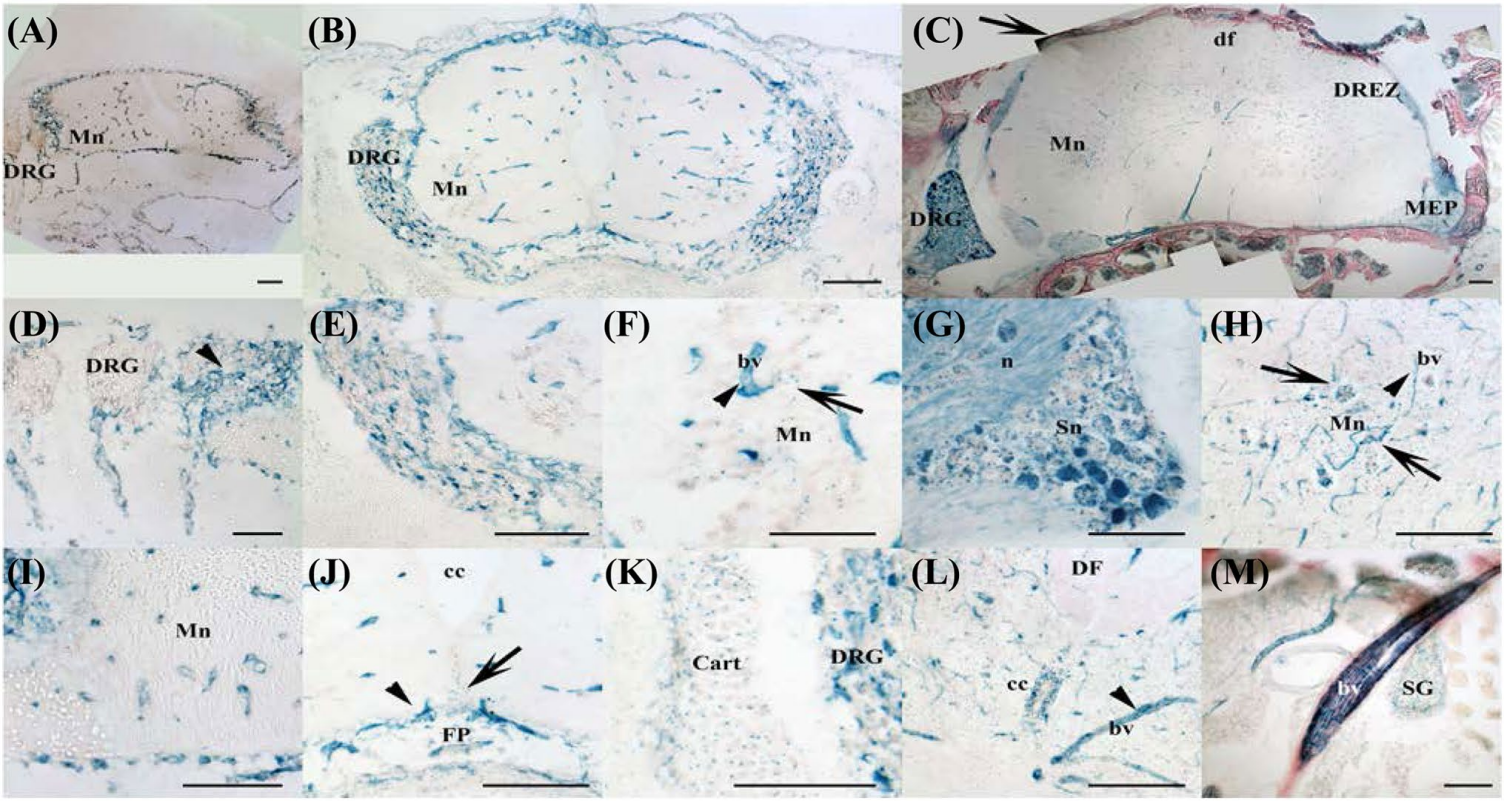
OSTF1 is widely expressed in the spinal cord and dorsal root ganglia (DRGs). **a**–**c** At E10, OSTF1 is found exclusively in the developing capillary network of the spine (panel **a**), surrounding OSFT1-negative DRGS (panel **b**) and motor neurons (panel **c**, Mn). **d**–**h** Strong expression in the blood vessels (bv and dark arrowheads) at E15. The neurons of the DRGs start expressing OSTF1 (panel **e**) at E15. It is at that stage that motor neurons (panel **f**) begin expressing OSTF1. **g** The floor plate (FP) but not the central canal (cc) express OSTF1. **h** The cartilage surrounding the spine expresses low levels of OSTF1. **i**–**m** Expression in the blood vessels (bv and dark arrowheads) in adult mice. **i, j** In the adult mouse, all DRG neurons express OSTF1, albeit at different levels, the signal is not restricted to the cell bodies and can be found in the neurites (n) of the dorsal roots to the Dorsal Root Entry Zone (DREZ) and the distal part of the dorsal funiculus (arrow in panel **i**, df) as well as at the Motor Exit Point (MEP). **k** Expression of OSTF1 in the motor neurons (arrows) is well established. **l** Likewise, OSTF1 is found in the central canal (cc). **m** Low level of OSTF1 is detected in the sympathetic ganglia (SG). Scale bars = 100 µm

In the spinal cord as in the brain, the capillary network was strongly LacZ positive as early as at E11, (Fig. 3a–c, arrowheads), at E14 and in adult tissues (Fig. 3d, i respectively). Noticeably, motor neurons expressed low levels of *Ostf1* from E14 until adulthood (Fig. 3b, e, k, arrows). At E14, the floor plate was observed to express low levels of *Ostf1* (Fig. 3g, FP) but this was no longer seen in the adult. In contrast, in the adult spine, the ependymal cells of the central canal were also LacZ positive (Fig. 3l, cc), while these structures were not LacZ positive in E14 and E11 spines (Fig. 3g, cc).

### Peripheral nervous system

Both trigeminal ganglia (Fig. 2o, arrow) and spiral ganglia (Fig. 2p, arrow) expressed high levels of OSTF1. While all the neurons of the spiral ganglia were LacZ positive at E14, expression was initially non-uniform in trigeminal neurons. In contrast, in the adult mouse, all neurons strongly expressed LacZ.

LacZ was detected in the sensory neurons of the Dorsal Root Ganglia (DRGs) from E14 onwards (Fig. 3a, b, d, e, respectively) when DRGS were composed of neurons expressing no, weak or strong OSTF1 expression. In adult DRGs, all neurons express *Ostf1* to some degree (Fig. 3i, j), indicating that *Ostf1* expression varies between different types of sensory neuron and their developmental stage. This was further supported by the mixture of LacZ positive and negative axons present in sensory nerves (Fig. 3J, n) near the DRGs and at Dorsal Root Entry Zone (DREZ) level (Fig. 3i, DREZ). Given the lateral position of the LacZ positive axon bundles in the dorsal funiculus (df), it appears likely that the DRG neurons which express high levels of *Ostf1* are nociceptors (Fig. 3i, arrow). In contrast, *Ostf1* signal in the sympathetic ganglia (Fig. 3m, SG) was weak, diffuse, and similar to that seen in the motor nerves at the Motor Exit Point (MEP, Fig. 3i), suggestive of a glial origin.

### Sensory organs—ear and eye

**Fig. 4:**
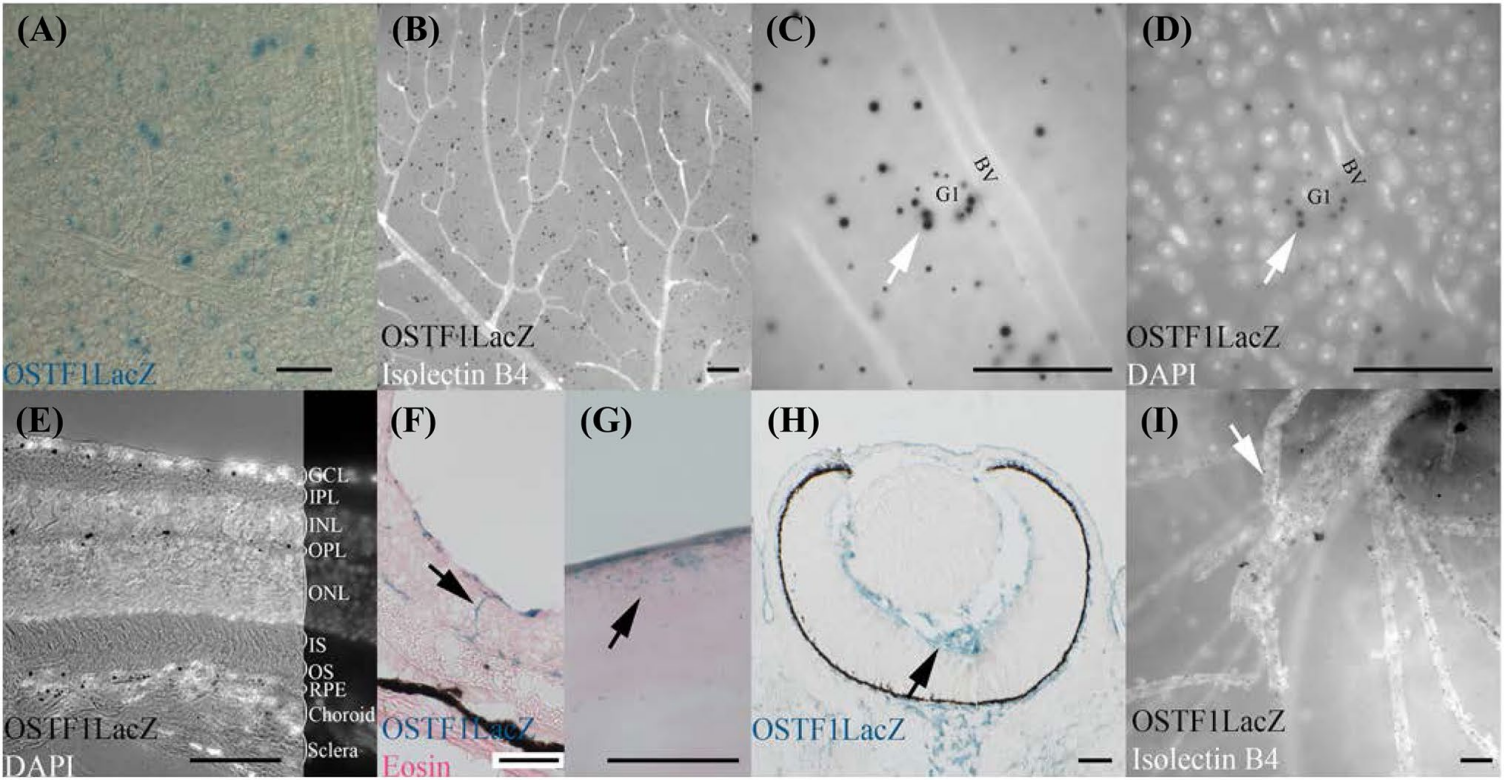
OSTF1 is expressed in the eye. **a**–**d** LacZ stained flat mounted retina indicate OSTF1 is scattered on the retina surface. **b, c** Isolectin-B4 staining (white in panel **b, c**) does not colocalise with OSTF1, suggesting that the retinal blood vessels (BV) do not express OSTF1. **c, d** OSTF1 is found concentrated, in the ganglia cell layer, around cells with large nuclei (stained with DAPI) and cytoplasm (arrow), presumably G1 cells. **e** In transverse section of the retina of an OSTF1^LacZ/LacZ^ albino mouse, OSTF1 is found in the ganglia cell layer (GCL), the outer plexiform layer (OPL) and the retinal pigmented epithelium (RPE) but not the inner plexiform layer (IPL), inner nuclear layer (INL), outer nuclear layer (ONL), inner (IS) and outer segments (OS) of the photoreceptors, choroid and sclera. **f** Muller glial cells (arrow) express OSTF1. **g** The outer cells of the cornea express OSTF1. **h, i** In the embryo and P6 pup, OSTF1 is found in the capillary network (arrows) and can be counterstained with isolectin B4 (white in panel **i**). All scale bars = 50 µm

In the ear, *Ostf1* was observed in the cochlear epithelium in the embryo (Fig. 2p, notched blue arrowhead) and also the adult (data not shown). *Ostf1* signal in the eye appears to be restricted to the retina. Striking differences in expression were observed between the developing and mature structures. In the developing eye and up until early postnatal stages when the retinal vasculature is completely remodelled (Pitulescu et al. 2010), *Ostf1* was exclusively found in the blood vessels (Fig. 4h, i). In contrast, in the adult retina, *Ostf1* was excluded from blood vessel networks lining the ganglia cell layer (Fig. 4a–d) and instead restricted to specific deeper layers (Fig. 4e, f) as well as outer layers of the cornea (Fig. 4g). Flat mounting of the retina revealed *Ostf1* expression on the eye’s inner surface was present in the ganglia cell layer (GCL, Fig. 4a, b). However, some signal was clustered around a few cells (Fig. 4c, arrow). Judging by their larger nuclei and somata (Fig. 4d, arrow), these were possibly G1 cells (Volgyi et al. 2009). In transverse section (Fig. 4e, f), LacZ was detected in GCL, in the outer plexiform layer (OPL), where horizontal cells reside, and in the retinal pigmented epithelium (RPE). In a few sections, we observed large columnar LacZ positive Muller glial cells connecting the GCL with the border between outer nuclear layer (ONL) and photoreceptor inner segment (IS) (Fig. 4f, arrow).

### Lung

**Fig. 5:**
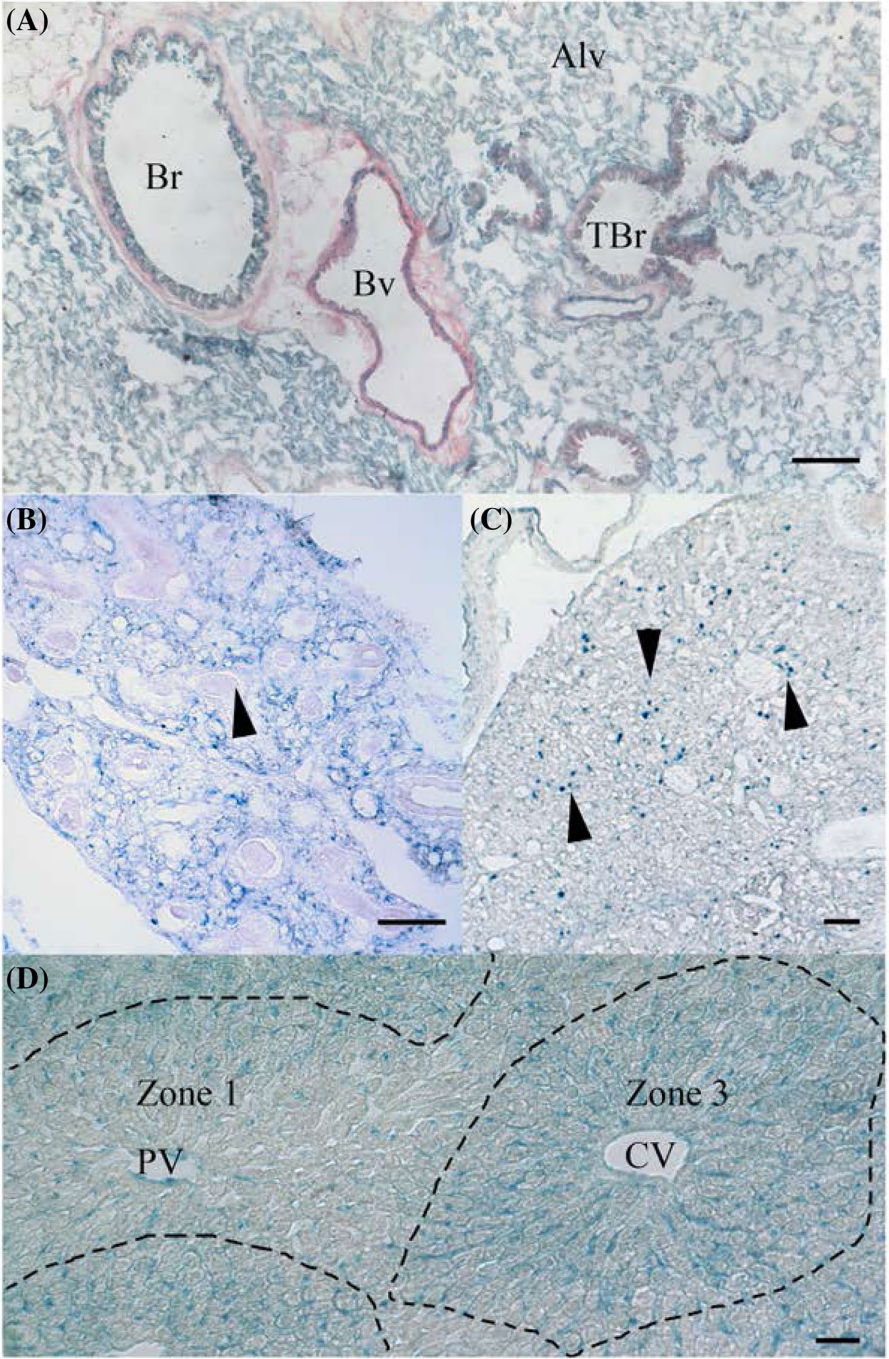
OSTF1 is expressed in lung and liver tissues. **a** In adult lung, OSTF1 is expressed in the alveolar parenchyma (Alv), the epithelium of bronchus (Br) and terminal bronchus (TBr) and blood vessel endothelium (Bv). **b** In embryonic lung (E15), OSTF1 is detected in the developing capillary network, but not in the bronchus (arrowhead). **c** In embryonic liver (E15), we find OSTF1 expression in distinct cells spread in the parenchyme, likely to be hematopoietic stem cells. **d** In contrast, in the adult liver, OSTF1 is found exclusively in endothelial cells in a low-to high gradient from the hepatic zone 1 surrounding the portal vein (PV) to zone 3 around the central vein (CV). Scale bars = 100 µm

X-Gal staining shows *Ostf1* expression in the embryonic lung at E14 and in the adult tissue. In adult lung, *Ostf1* appears ubiquitous in the respiratory epithelium of the alveolar parenchyme (Fig. 5a, Alv). It is also found in the epithelium of the bronchi (Br) and of the terminal bronchi (TBrl). As in most tissues, OSTF1 is found in the endothelial cells of blood vessels (Bv), according to vessel morphology both arterioles and venules. In contrast, in the embryo, *Ostf1* localises to the growing network of blood vessels but is excluded from the bronchi (Fig. 5b, arrow).

### Liver

Expression of *Ostf1* changes dramatically between embryonic and adult liver (Fig. 5c, d). In the embryo, *Ostf1* is strongly expressed in cells scattered around the liver (Fig. 5c, arrows), which may represent hematopoietic stem cells, but it is absent from the remainder of the organ. In contrast, in adult liver, *Ostf1* is absent from the hepatocyte cordons, but strongly expressed in the sinusoidal endothelial cells that surround the central veins (Fig. 5d, dashed lines, Cv). Interestingly, compared to the area surrounding the central vein, *Ostf1* expression surrounding the portal vein is weaker and limited to a subset of endothelial cells (Fig. 5d, PV). This is suggestive of a gradient from low-to-high *Ostf1* expression from hepatic acinus zone 1–3, which is accompanying blood flow.

### Heart

**Fig. 6:**
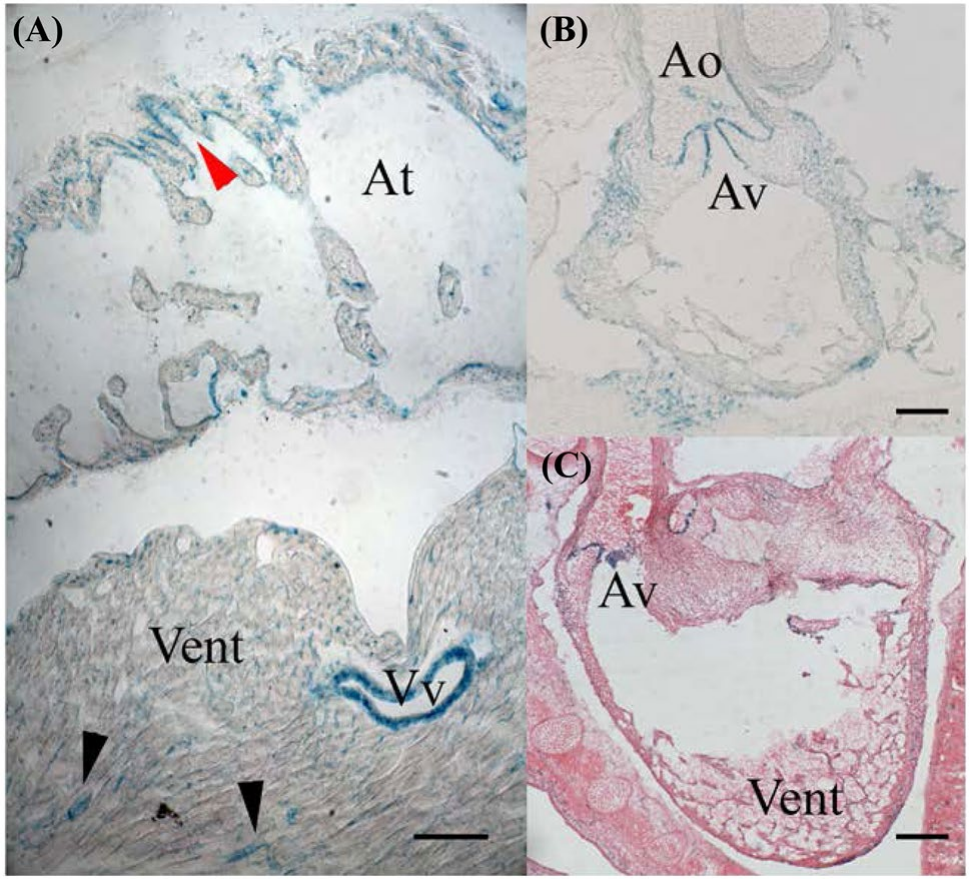
OSTF1 is expressed in the adult and embryonic heart. **a** In the adult heart, OSTF1 is found in the endothelium (red arrow) of the atrium (At), the large (Vv) and small vessels (arrowheads) that form the vasa vasorum of the ventricle (Vent). Small puncta indicate that the cardiomyocytes also express OSTF1. **b, c** In embryos, OSTF1 expression is seen in the aortic valves (Av) and the endothelium of the aorta (Ao) but not in the cardiac endothelium itself. Scale bars = 100 µm

In the adult heart, *Ostf1* was found to be expressed in the endothelium of the atrium (Fig. 6a, red arrowhead in At) and the vasa vasorum of the ventricle (Fig. 6a, Vv, black arrowheads and Vent) but surprisingly it was not found lining the inside of the ventricles (data not shown). Some heart myocytes and pericardial cells expressed low levels of *Ostf1* as seen by small X-Gal puncta (Fig. 6a). In embryonic heart at E14, *Ostf1* was found to be expressed at the surface of the lips of the aortic valve (Fig. 6b, c, Av), as well as the endothelium of the aorta (Fig. 6b, Ao).

### Kidney

**Fig. 7:**
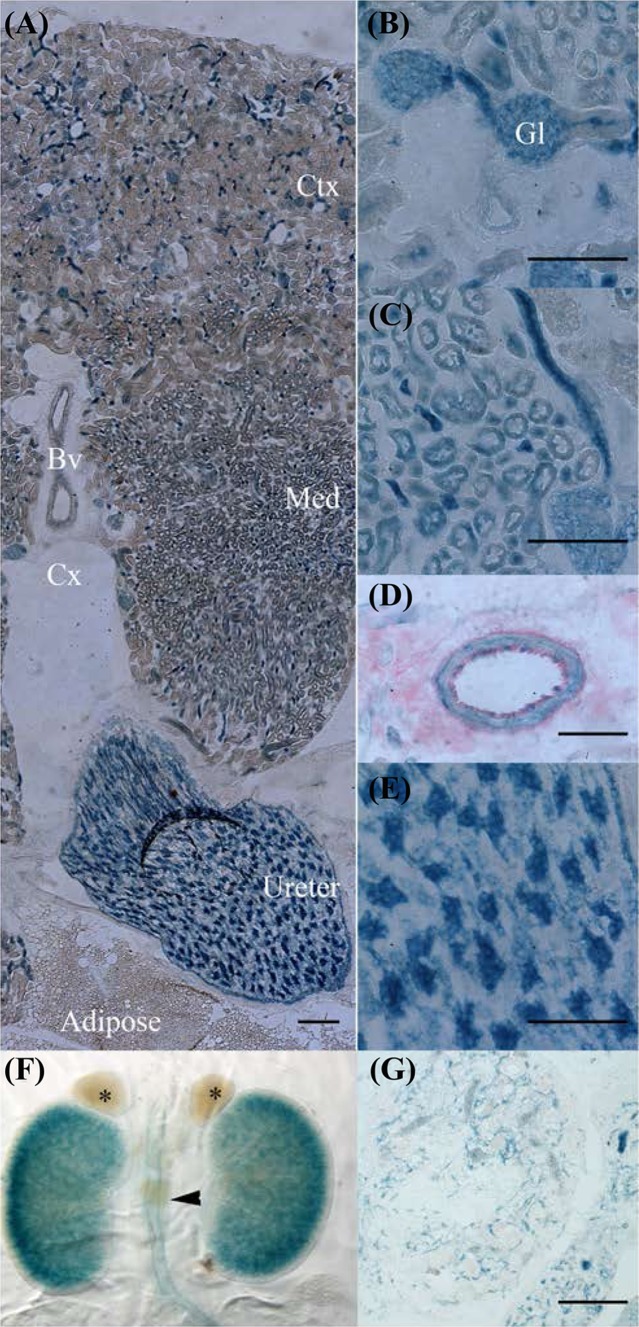
OSTF1 is highly expressed in the kidney. **a** Transverse section through adult kidney demonstrates that OSTF1 is expressed in the cortex (Ctx), Medulla (Med), blood vessels (Bv), calyx (Cx) and Ureter but not in the underlying adipose tissue. **b** In the cortical region, OSTF1 is found in the glomeruli (Gl) and the proximal convoluted tubules. **c** OSTF1 is expressed at varying levels in all tubules of the medulla. **d** Renal arteries express OSTF1 in the endothelium and underlying smooth muscle. **e** The ureter wall expresses OSTF1 in a mosaic of high expression patches surrounded by low expression. **f** Whole mount expression in the P0 kidney shows wide expression in the kidneys and ureter (arrowhead) but not the adrenal glands (*). **g** In the embryonic kidney, OSTF1 is exclusively confined to the developing capillary network. Scale bars = 100 µm

*Ostf1* was widely expressed in the kidney, from cortex to ureter (Fig. 7a). In the cortex, *Ostf1* was found in the glomeruli (Fig. 7b, Gl) and associated blood vessels and the proximal convoluted tubule. In the medulla (Fig. 7c), *Ostf1* was found to be expressed to varying degrees in all tubules, with highest expression in the proximal convoluted tubules. In both regions, blood vessels were LacZ positive (Fig. 7d) both in the endothelium and also in the underlying smooth muscle rings. The renal papilla clearly exhibited the highest level of *Ostf1*expression in the kidney (Fig. 7a, e). Kidney *Ostf1* expression clearly begins early with LacZ being detected throughout the kidney at P0 except for the outer nephrogenic zone (Fig. 7f) and in blood vessels of the developing organ at E14 (Fig. 7g). *Ostf1* expression was markedly absent from the adrenal glands at this timepoint (Fig. 7f, asterisks).

### Placenta

**Fig. 8:**
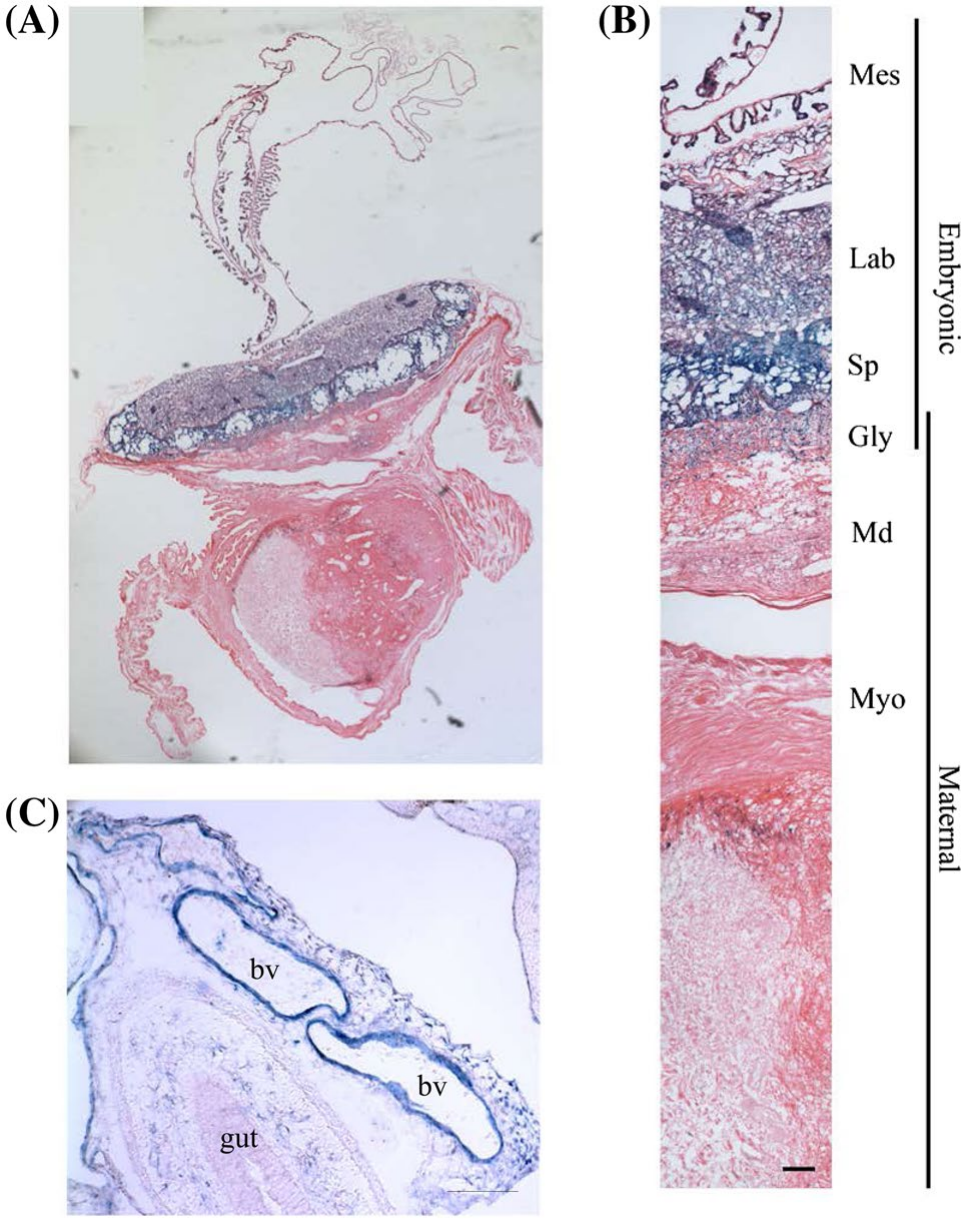
OSTF1 is expressed throughout the embryonic placenta and the umbilical cord. **a** Low magnification view of the placenta of an *Ostf1* ^*LacZ*/*LacZ*^ embryo implanted in the uterine wall of its *Ostf1* ^+/*LacZ*^ mother. **b** All tissues of embryonic origin express high levels of OSTF1, specially the spongiotrophoblast layer (Sp) and the labyrinth layer (Lab). The glycogen cells also express high levels of OSTF1 while less is found in the maternal decidua (Md) and none in the myometrium (Myo). There is high level of expression in the mesodermal cells of the yolk sac (Mes). **c** In the umbilical cord, there is high level of expression in the blood vessels (bv) but none in the gut. Scale bar **a** = 500 µm, scale bars in **b, c** = 100 µm

**Fig. 9:**
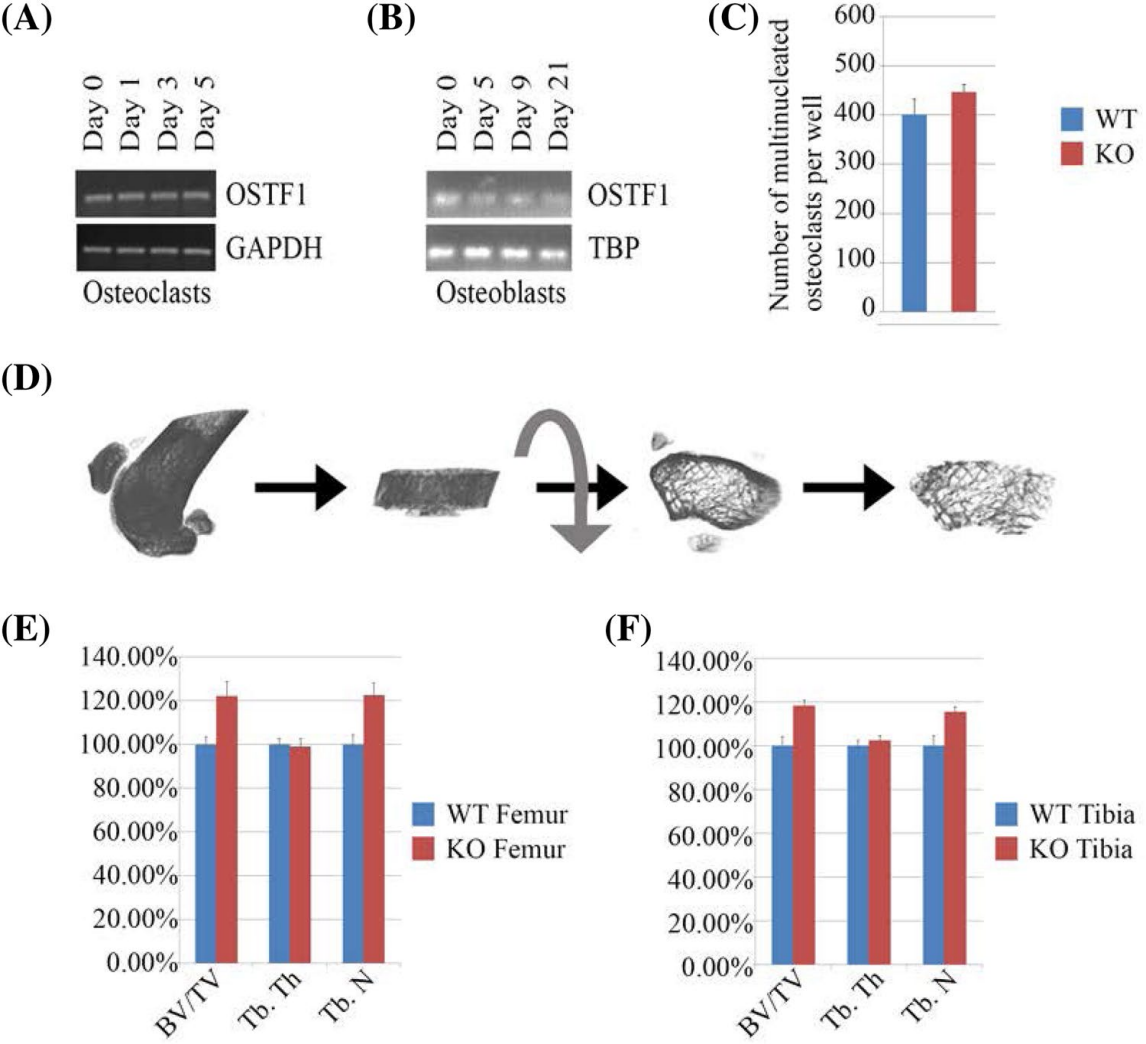
RT PCRs indicate OSTF1 is expressed in osteoclasts and osteoblasts and its loss leads to increased bone density. **a** *Ostf1* mRNA is detected in differentiating osteoclast cells from primary bone marrow-derived macrophages at day 0 until fully differentiated at day 5. GAPDH was used as a positive control. **b** *Ostf1* mRNA is also expressed throughout osteoblasts differentiation. TBP was used as a positive control. **c** Loss of *Ostf1* expression does not perturb osteoclast differentiation. Equal numbers of primary bone marrow-derived macrophages from wild type and (WT) *Ostf1* ^*LacZ*/*LacZ*^ (KO) male mice were plated and differentiated to osteoclast in vitro (*n* = 3 *for each genotype*). Mature osteoclasts were identified and quantified following TRAP staining (*n.s*. not significant). **d** Micro-CT scanning procedure from bone scanning to selection of trabecular bone for analysis. **e** Femurs from adult male *Ostf1* ^*LacZ*/*LacZ*^ mice (*n* = 14) exhibit elevated bone density (BV/TV *p* ≤ 0.01, asterisk) and trabecular number (Tb.N *p* ≤ 0.01, *asterisk*) compared to wild type (WT—*n* = 16) as ascertained by µCT scanning. No difference in trabecular thickness (Tb.Th *p* > 0.2, *n.s*. not significant) was observed. **f** Tibia from adult male *Ostf1* ^*LacZ*/*LacZ*^ mice (*n* = 12) exhibit elevated bone density (BV/TV *p* < 0.01, asterisk) and trabecular number (Tb.N *p* < 0.01, asterisk) compared to wild type (WT—*n* = 16) as ascertained by µCT scanning. No difference in trabecular thickness (Tb.Th *p* > 0.2, *n.s*. not significant) was observed

All the embryo-derived parts of the E14 placenta were found to be LacZ positive (Fig. 8a, b); this applied particularly to the labyrinth and spongiotrophoblast (Fig. 8b, Lab and Sp). This may in part be due to the large amount of vasculature in that region. In the umbilical cord, the highest level of expression was observed in the endothelial lining of the blood vessels (Fig. 9c, Bv), but no expression was found in the underlying gut structure (Fig. 9c, gut).

### Expression in bone tissue

**Fig. 10:**
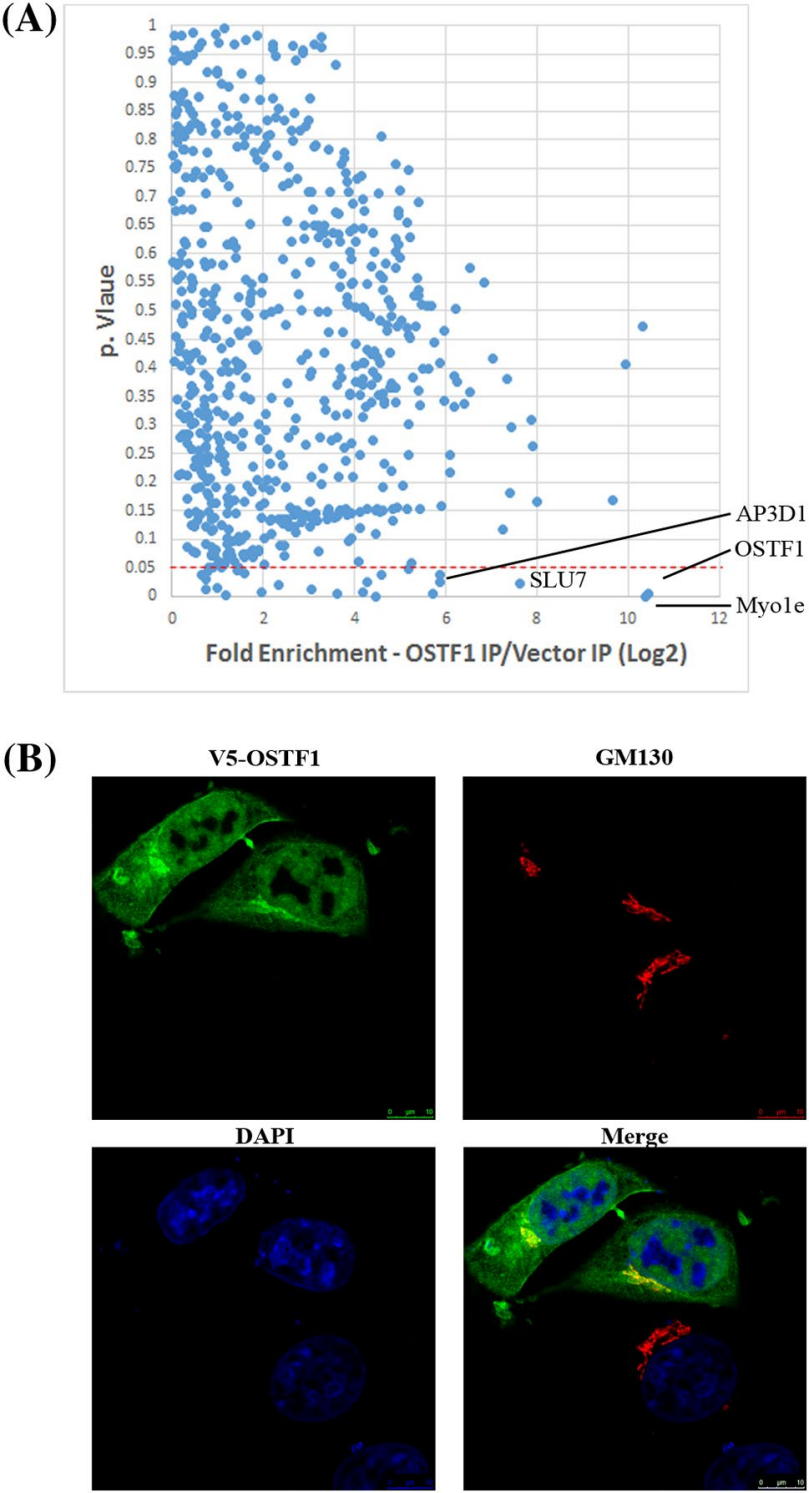
Identification of OSTF1-interacting proteins. **a** Volcano plot showing proteins identified by LC-MS/MS that are enriched in V5-OSTF1 IPs. HEK293 cells were transiently transfected in triplicate with empty vector or V5-OSTF1. Lysates were subjected to V5 IP and co-immunoprecipitating proteins identified by LC-MS/MS. X-axis shows average fold enrichment of individual proteins (blue points) in cells expressing V5-OSTF1 compared to empty vector. Y axis shows *p* value. Dashed red line represents *p* = 0.05 cutoff. **b** OSTF1 localises to the cytosol, nucleus and golgi. V5-OSTF1 was transiently transfected into HEK293 cells, and cells were fixed, permeabilised and immunostained with anti-V5 antibody (green), anti-GM130 (red) to stain the golgi, and DAPI (blue) to label the nucleus. Scale bar = 10 µm

Given that OSTF1 is thought to impact osteoclast activity (Reddy et al. 1998), its identification in bones was seen as a priority. In embryonic tissue, the pre-existing cartilage matrix that will develop into bone was only LacZ positive in *Ostf1* ^+/*LacZ*^ and *Ostf1* ^LacZ/LacZ^ embryos (Fig. 3k, Cart; Fig. 2n, arrows pointing to lower jaw and facial bone primordium), but not their wild-type siblings, suggestive of *Ostf1* expression in bone. More developed bone tissues harbour endogenous beta-galactosidase-like activity, in particular in osteoclasts (Kopp et al. 2007), making it problematic to draw conclusion from X-Gal staining of developing or indeed established bones. We, therefore, performed semi-quantitative RT-PCR of cultured osteoclast and osteoblasts as they differentiated from bone marrow to identify whether they expressed *Ostf1. Ostf1* expression was detected at all stages of differentiation of both cell types. However, while *Ostf1* appeared to be evenly expressed in osteoclasts throughout differentiation, its expression in osteoblasts decreased over time (Fig. 10a, b).

### Phenotyping of the *Ostf1*^*LacZ*/*LacZ*^ mouse

We have generated a mouse that is knocked out for *Ostf1*. In absence of any clear developmental phenotype, and in order to define where, when and what could be perturbed by loss of *Ostf1* expression, we have characterised the expression of *Ostf1* in embryonic and adult mouse. In parallel to the LacZ staining, we have analysed the histology of the various tissues analysed by routine haematoxylin and eosin staining and observed no significant histological abnormalities. It is, however, possible that the physical or functional abnormalities may not show up by simple histology.

### Bone phenotyping

OSTF1 was first described as an indirect osteoclast stimulating factor. We, therefore, compared the differentiation of *Ostf1* ^LacZ/LacZ^ osteoclasts with wild-type controls. Following differentiation in vitro, primary bone marrow-derived osteoclasts were stained for Tartrate Resistant Acid Phosphatase (TRAP) to permit quantitation of osteoclast differentiation. No difference was observed in the ability of primary bone marrow-derived macrophages to differentiate into TRAP-positive osteoclasts between wild-type (400.5/Well, SEM 32.1, Fig. 9c) and *Ostf1* ^*LacZ*/*LacZ*^ (447/well, SEM 15.7, *p* = 0.21) cultures. Thus, OSTF1 is not required for osteoclast differentiation in vitro.

We next investigated whether loss of *Ostf1* might impact on osteoclast activity, and analysed bone density by micro-CT scanning, focussing on tibiae and femurs of age-matched knockout and wild-type male mice (15 weeks; *n* = 8 WT, 6 KOs, both legs scanned). This method, which relies on X-ray imaging of a rotating bone, reveals the fine trabecular bridges located under the growth plate without destroying the bone. Images were reconstructed and a volume of interest located just under the growth plate was computationally analysed for bone volume, density and surface (Fig. 9d). This revealed that *Ostf1* ^LacZ/LacZ^ tibia and femur were significantly denser than wild-type counterparts. Normalised to wild type, the *Ostf1* ^LacZ/LacZ^ bone volume/tissue volume (BV/TV) was 122% (SEM 6.5%, *p* < 0.01) for the femur and 118.43% (SEM 2.33%, *p* < 0.01) for the tibia. Whilst trabecular thickness of WT and *Ostf1* ^LacZ/LacZ^ bones was indistinguishable, the trabecular number was increased in *Ostf1* ^LacZ/LacZ^ to 122.52% (SEM 5.49%, *p* < 0.01) in the femur and to 115.41% (SEM 2.31%, *p* < 0.01) in the tibia (Fig. 9e, f and Supplemental Fig. 2). We concluded that loss of OSTF1 confers mild osteopetrosis-like phenotype caused by an increase in trabecular number but not thickness.

### OSTF1 interacting proteins

In order to understand how OSTF1 might influence bone density, a proteomic approach was used to identify OSTF1-interacting proteins in HEK293 cells, the cell line which was first used to demonstrate that OSTF1 stimulated secretion of a factor/s that promoted osteoclast differentiation (Reddy et al. 1998). HEK293 cells were transfected in triplicate with either empty vector (Vector) or a vector encoding human OSTF1 tagged at the N-terminus with a V5-epitope tag (V5-OSTF). Cell lysates were then subjected to V5 immunoprecipitation and co-immunoprecipitating proteins identified by LC-MS/MS.

This approach identified 18 proteins that were significantly enriched (*p* < 0.05) in IPs from cells expressing V5-OSTF1 compared to vector control (Fig. 10a; Table 2). This list contained both cytosolic and nuclear-localised proteins which were reflected by the subcellular localisation of V5-OSTF1 in transfected HEK293 cells. Immunofluorescence demonstrated V5-OSTF1 to be localised to the cytosol, the golgi and the nucleus, but excluded from regions of dense chromatin (Fig. 10b). Reassuringly, the top interacting protein was Myosin 1e, which has been previously identified to interact with OSTF1, and is thought to regulate cytoskeletal reorganisation and vesicular trafficking (Tanimura et al. 2016). Interestingly, Myosin 1e has also been demonstrated to regulate secretion of the chemokine CCL2, deficiency of which causes increased bone mass in mice (Sul et al. 2012; Wenzel et al. 2015). An additional OSTF1-interacting protein was found to be AP3D1, a subunit of the golgi-localised AP3 vesicular trafficking complex, disruption of which leads to dysregulation of peptide hormone release in mice (Sirkis et al. 2013). The majority of the remaining interacting proteins are associated with nuclear functions including RNA splicing, chromatin remodelling and gene expression (Table 2). A notable absence was SMN1, although this may reflect the cell type used in the immunoprecipitation experiment. However, many of the OSTF1-interacting proteins identified function in RNA splicing, where SMN1 facilitates the assembly of snRNP spliceosome components.

**Table 2:** Table showing the identity, function, disease association, fold enrichment and *p* value of proteins identified as co-immunoprecipitating with V5-OSTF1 from HEK293 cells

Gene symbol	Full name	Function	Disease associations	Log2 fold enrichment	Actual fold enrichment	*p* Value
OSTF1	Osteoclast stimulation factor 1	Cytoskeleton	Deleted in microdeletion sydrome with epilepsy and intellectual disability	10.4	1391.1	0.003478529
MYO1E	Myosin 1E	Vesicular trafficking; actin reorganisation	Mutated in focal segmental glomerulosclerosis-6	10.4	1319.5	0.000231218
SLU7	SLU7 homolog	RNA splicing		7.6	196.2	0.023058311
AP3D1	Adaptor-related protein complex 3, delta-1 subuint	Vesicular trafficking	Mutated in Hermansky-Pudlak syndrome and "mocha" mose model	5.9	58.1	0.024118142
ACIN1	Apoptotic chromatin condensation inducer 1	Apoptotic chromatin condensation; RNA splicing		5.9	58.1	0.038838599
GTPBP4	GTP-binding protein 4	Unknown		5.7	52.6	0.005445883
TOP2B	Topoisomerase (DNA) II beta	Transcription; DNA replication; chromatin remodelling		5.2	36.5	0.047338493
DNTTIP2	Deoxynucleotidyltransferase terminal interacting protein 2	Transcription; chromatin remodelling		4.6	23.9	0.037307658
SCAF11	SR-related CTD associated factor 11	RNA splicing		4.5	22.2	0.000497072
SNIP1	Smad nuclear interacting protein 1	Transcription	Mutations cause PMRED (psychomotor retardation, epilepsy, and craniofacial dysmorphism)	4.3	19.4	0.024277978
UPF3B	UPF3B, regulator of nonsense mediated mRNA decay	mRNA surveillance; nonsense mediated decay	Mutations in XLID (X-linked intellectual disability) with autism, ADHD and scizophrenia	4.2	18.0	0.007231004
FAM98B	Family with sequence similarity 98 member B	RNA transport		3.6	12.3	0.00373743
PAK1IP1	PAK1 interacting protein 1	Ribosomal stress response	Mutated in mouse model of orofacial clefting	3.0	8.2	0.011385542
SS18	SS18, nBAF chromatin remodelling complex subunit	Transcription	Chromosomal translocation causes synovial sarcoma	2.3	5.1	0.019649035
YTHDC1	YTH domain containing 1	RNA splicing		2.0	3.9	0.006920967
RNPS1	RNA binding protein with serine rich domain 1	RNA transport; nonsense mediated decay		1.6	3.0	0.040180758
FTSJ3	FTSJ3	rRNA processing		1.4	2.7	0.04653522
FIP1L1	Factor interacting with PAPOLA and CPSF1	RNA polyadenylation	Interstitial chromosomal deletion results in fusion to the PDGFRA gene in chronic eosinophilic leukaemia	1.2	2.3	0.002115712

### Behaviour phenotyping

Although we did not identify SMN as an OSTF-interacting protein in HEK293 cells, this interaction may still occur in other cell types such as motor neurons. OSTF1 was previously reported to interact with SMN (Kurihara et al. 2001), loss of SMN impacts bone development (Shanmugarajan et al. 2007) and SMN is expressed by osteoclasts (Kurihara et al. 2001), we wondered if the *Ostf1* ^*LacZ*/*LacZ*^ would share phenotypes seen in SMN mutant mice. SMN knockout mice are embryonic lethal (Schrank et al. 1997) and hypomorphs suffer from muscle weakness that inversely correlates with the level of SMN expression (Edens et al. 2015; Lefebvre et al. 1997). This is clearly not the case of the *Ostf1* mice which live up to a year without obvious phenotype but it was still possible that *Ostf1* mice behave abnormally.

**Fig. 11:**
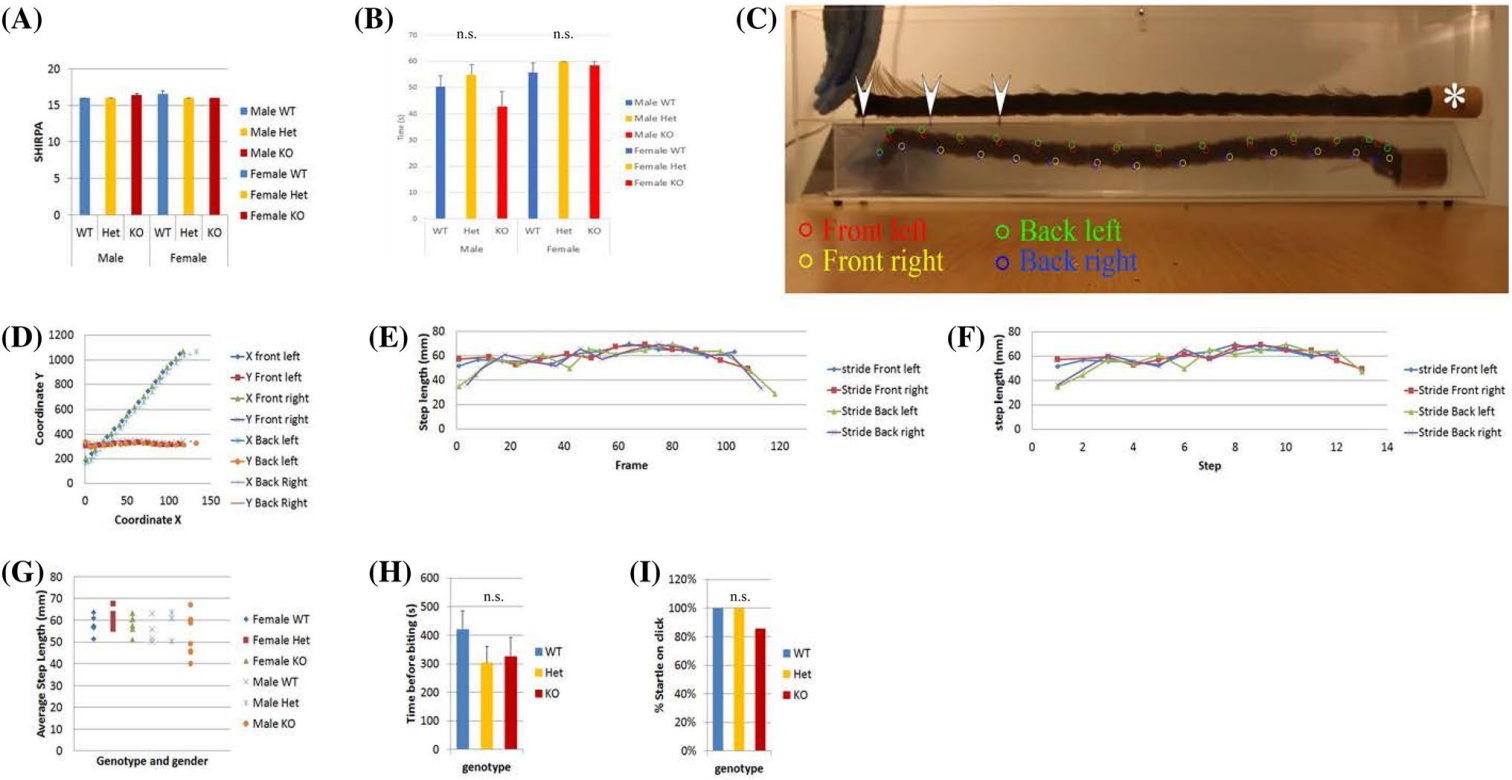
*Ostf1* ^*LacZ*/*LacZ*^ mice behave normally. **a** No difference is observed between wild type, *Ostf1* ^+/*LacZ*^ and *Ostf1* ^*LacZ*/*LacZ*^ mice is SHIRPA test. **b** No difference is observed between wild type, *Ostf1* ^+/*LacZ*^ and *Ostf1* ^*LacZ*/*LacZ*^ mice in hang wire latency (WT male *n* = 4, Het male *n* = 4, KO male *n* = 8, WT female *n* = 7, Het female *n* = 7, KO female *n* = 6). **c**–**f** Gait analysis indicate that *Ostf1* ^*LacZ*/*LacZ*^ walk normally. **c** Representative flattened timelapse of a mouse walking through the cat walk box from being placed in the box (left, where the gloved hand is) to the relatively darker side where a cardboard tube is (asterisk). Each paw step is labelled with a coloured circle (front left, red; front right, yellow; back left, green; back right, blue). The three bars, pointed by white arrowheads, are at 10, 20 and 30 cm each from the side of the box and are used for step calibration. **d** Tracking of each paw as the mouse walks provides a set of coordinates showing that the mouse has walked without pause in a straight line. **e** From these coordinates, step length can be inferred and plotted against frame, showing coordination of front left and back right paws and front right and back left. **f** Likewise, step length plotted against step indicates that each paw moves regularly. **g** Average step size in millimetre for each animal tested indicate that there is no significant difference between genotype. **h** Mice show no difference in chocolate burrowing test indicating normal olfaction (*n.s*. not significant). **i** Mice show no difference in Preyer’s auditory reflex (WT *n* = 11, Het *n* = 11, KO *n* = 14, WT vs. Het *p* > 0.14, WT vs. KO *p* > 0.25, *n.s*. not significant)

As many of the OSTF1-interacting proteins (Table 2) result in neurological defects and human microdeletion patients where the *OSTF1* gene is one of six deleted genes also display neurological abnormalities, *Ostf1* mice were rigorously examined via a SHIRPA protocol, a battery of non-intrusive tests where mice are observed mostly unchallenged (Rogers et al. 1997). These tests are designed to monitor neuropsychiatric, cerebellar and sensory functions together with muscle function. *Ostf1* ^LacZ/LacZ^ mice had normal SHIRPA score (Fig. 11a). Part of the SHIRPA protocol involves balance and coordination tests where mice are put on a grid, the grid is suddenly inverted and lightly shaken and time taken for mice to release their grip recorded. The *Ostf1* ^LacZ/LacZ^ cohort showed no difference than control siblings (Fig. 11b). Next, gait of wild type and mutant mice were examined using a cat walk box, a transparent box with a translucent bottom where paw position can be observed (Fig. 11c). Mice were filmed running for one metre towards the relative shade of an empty cardboard tube. Movies were analysed by recording paw print using a custom imageJ plug-in. Each paw strike gave a coordinate that could then be plotted (Fig. 11d) and from which step size was calculated. Thus, plotting against frame allows analysis of paw strike synchrony (Fig. 11e), and plotting against step allows to see if the run is regular (Fig. 11f). Finally, by averaging the step for each paw for each animal, it was possible to see if there was a difference in step size between wild type, *Ostf1* ^LacZ/+^ and *Ostf1* ^LacZ/LacZ^. Analysis of the movies did not reveal any difference between cohorts: all animals had regular synchronic gait and normal step size, (Fig. 11g).

*Ostf1* is expressed in the hippocampus and the mammillary bodies, which are thought to be involved in olfactory memory (Yokosuka et al. 1999). We tested our mice for olfaction defects by habituating them to chocolate then introduced them to a clean cage where chocolate was hidden under bedding (Yang and Crawley 2009). Mice were timed to find the chocolate up to a maximum of 10 min. No difference between cohorts was observed in these tests (Fig. 11h).

Since there is significant expression of *Ostf1* in the inner ear epithelium and in the cochlear ganglion (Fig. 2p) mice were examined for signs of auditory defects by testing with a click box. Briefly, a small box produces a loud click and causes a flick of the ears [Preyer’s reflex (Jero et al. 2001)] and a startle response characterised by neck contraction. Similar to the olfaction tests, no difference was observed in the auditory response between cohorts (Fig. 11i).

## Discussion

OSTF1 was previously described as a cytoplasmic protein with SH3 and ankyrin domains that indirectly activates osteoclast differentiation and regulates their bone-resorbing activity (Reddy et al. 1998). OSTF1 was suggested to bind to a number of other proteins and previous studies suggested functions in spinal motor neuron atrophy and a microdeletion syndrome (9q21.13) (Baglietto et al. 2014; Boudry-Labis et al. 2013; Kurihara et al. 2001; Lu et al. 2006; Szymkiewicz et al. 2004; Vinayagam et al. 2011). To our knowledge, we have generated the first *Ostf1* mouse model. We show that this is a functional *Ostf1* knockout, with LacZ placed under control of *Ostf1’s* regulatory elements. The *Ostf1* ^*LacZ*/*LacZ*^ mice were fertile and without overt deleterious phenotype, at least in the absence of challenge. Significantly, none of the phenotypes observed in patients with the *OSTF1*-encompasing microdeletion syndrome were observed in either *Ostf1* ^*LacZ*/+^ *or Ostf1* ^*LacZ*/*LacZ*^ mice, suggesting that heterozygous loss of *OSTF1* is not causal of the pathogenesis of the syndrome in mice. However, it is possible that heterozygous loss of *OSTF1* may modify the phenotype of loss of other genes within the 9q21.13 locus.

As expected, *Ostf1* was found to be expressed in osteoclasts. However, while loss of *Ostf1* does not impair osteoclast differentiation, it has a mild effect on bone architecture since both tibia and femur of *Ostf1* ^LacZ/LacZ^ have a higher BV/TV than normal due to increase in trabecular number, but not thickness. Elucidating whether this is an effect on decreased osteoclastic activity or increased osteoblastic activity—or possibly both- will need to be further investigated. Since we and others have shown that OSTF1 interacts with Myosin 1e (which can regulate secretion of CCL2/MIP1) and AP3D1 (which regulates peptide hormone release) it will be interesting to investigate circulating hormones in *Ostf1* knockout mice.

OSTF1 has been reported to interact with SMN (Kurihara et al. 2001), a spinal muscular atrophy gene. Like SMN, OSTF1 is expressed in osteoclasts and motor neurons. However, we do not find embryonic lethality or rapid wastage associated with loss or downregulation of SMN (Lefebvre et al. 1997) in the *Ostf1* knockouts. Indeed, *Ostf1* ^lacZ/LacZ^ behaves normally and live up to 1 year without showing signs of muscle wastage. It is, therefore, unclear if the OSTF1–SMN interaction originally found by yeast two hybrid and pull down of cell lysate (Kurihara et al. 2001) occurs in vivo.

Taking advantage of the LacZ cassette, we undertook an in-depth analysis of *Ostf1* expression in both embryonic and adult mice. While various electronic resources are available that provide expression analysis, they often lack detailed description and are generally limited to tissue level. We show here that *Ostf1* is expressed widely in multiple organs, tissues and cell types, including the brain where *Ostf1* expression was somewhat controversial (Baglietto et al. 2014; Boudry-Labis et al. 2013). Interestingly, our results indicate *Ostf1* is widely expressed in the vascular compartment (endothelial cells). However, *Ostf1* is not found in all blood vessels, and throughout development, suggestive of a specialised function in a subset of vessels. Perhaps the most striking vascular expression of *Ostf1* was observed in the specialised vasculature of the liver. It will be interesting to test the functional relevance of this in the future, for instance by analysing angiogenesis and/or regeneration following liver damage. More complex still but not less striking is Ostf1 expression in the kidney, where it was found to be distributed to a series of specialised structure including the vasculature, the glomeruli and the nephrons. Given that unchallenged *Ostf1* ^LacZ/LacZ^ mice do not show and physical or histological signs of kidney disease, it will be interesting to challenge this organ too, to elucidate the functional relevance of this fascinating distribution.

## Electronic supplementary material

Below is the link to the electronic supplementary material.

**Supplemental Figure 1**: Absence of blue X-Gal precipitate following LacZ staining on wild type tissue demonstrates the specificity of beta Galactosidase activity. We find no staining in the spinal cord (A), including the motor neurons (B), the brain (C), adult retina (D), E14 embryonic retina (E), liver (F), lung (G), including bronchi and blood vessels (H), heart (I), cortical and medular kidney (J, K respectively), even at high magnification (L). There is no signal in E11 or E14 embryos (M, N respectively), nor in the E14 placenta (O), adult spleen (P) and pancreas (Q). All scale bars = 100 µm except C, M, N, O where scale bars = 1mm. (PDF 100 KB)

**Supplemental Figure 2: Box and whisker plots showing micro CT results**. Error bars = +/- SEM. For Tibia measurements, WT n=16 and *KO* n=12. For Femur measurements WT n=16 and *KO* n=14. (PDF 65 KB)
