# Supplementary figures and images for: Osteoclast stimulation factor 1 (Ostf1) KNOCKOUT increases trabecular bone mass in mice
Source: Mamm Genome. 2017 Sep 21;28(11):498–514. doi: 10.1007/s00335-017-9718-3 (PMC5680368; doi:10.1007/s00335-017-9718-3)

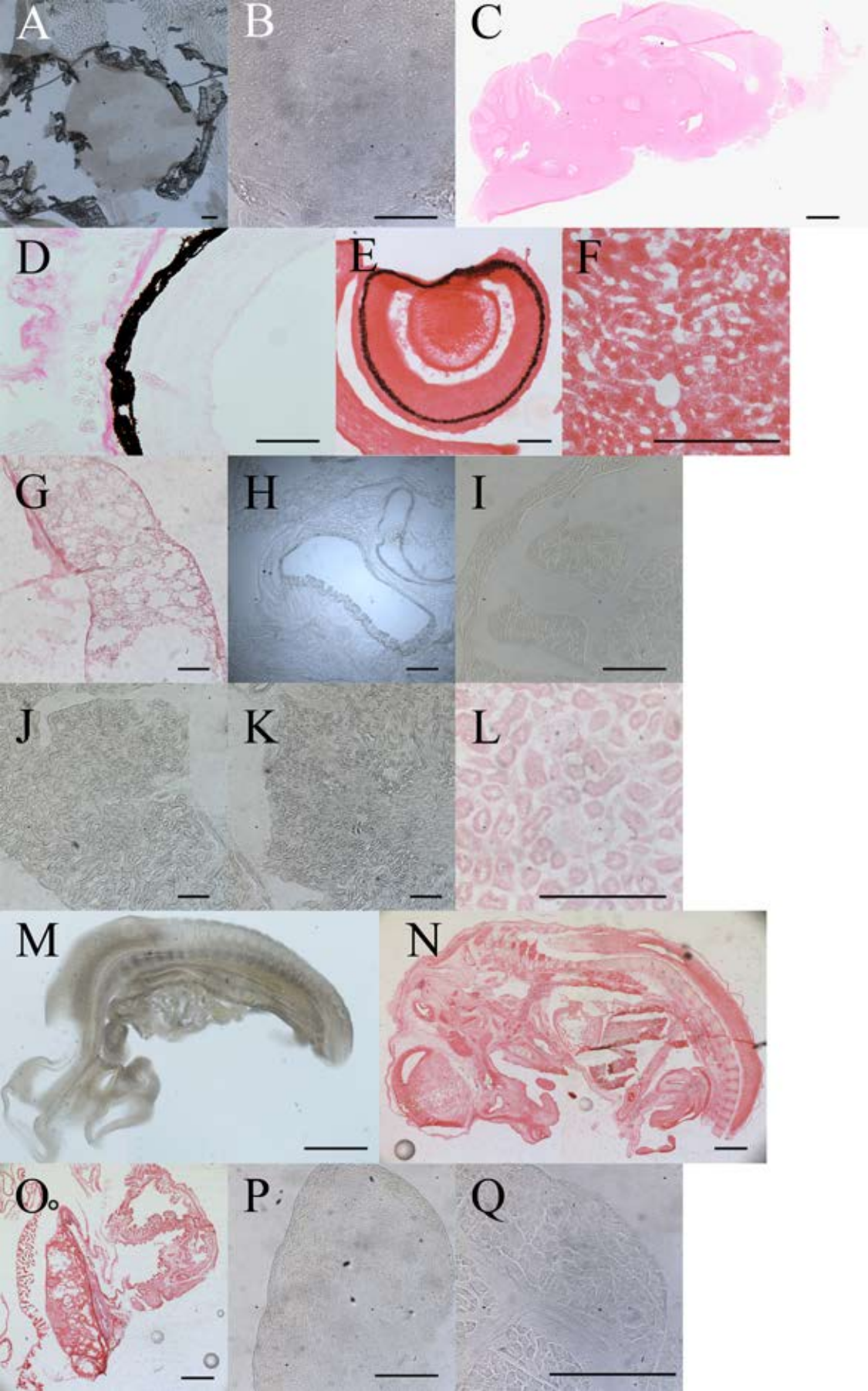

Supplement: Supplementary file 1 — Supplemental Figure 1: Absence of blue X-Gal precipitate following LacZ staining on wild type tissue demonstrates the specificity of beta Galactosidase activity. We find no staining in the spinal cord (A), including the motor neurons (B), the brain (C), adult retina (D), E14 embryonic retina (E), liver (F), lung (G), including bronchi and blood vessels (H), heart (I), cortical and medular kidney (J, K respectively), even at high magnification (L). There is no signal in E11 or E14 embryos (M, N respectively), nor in the E14 placenta (O), adult spleen (P) and pancreas (Q). All scale bars = 100 µm except C, M, N, O where scale bars = 1mm. (PDF 100 KB) [file 335_2017_9718_MOESM1_ESM.pdf]

# Normalised Data

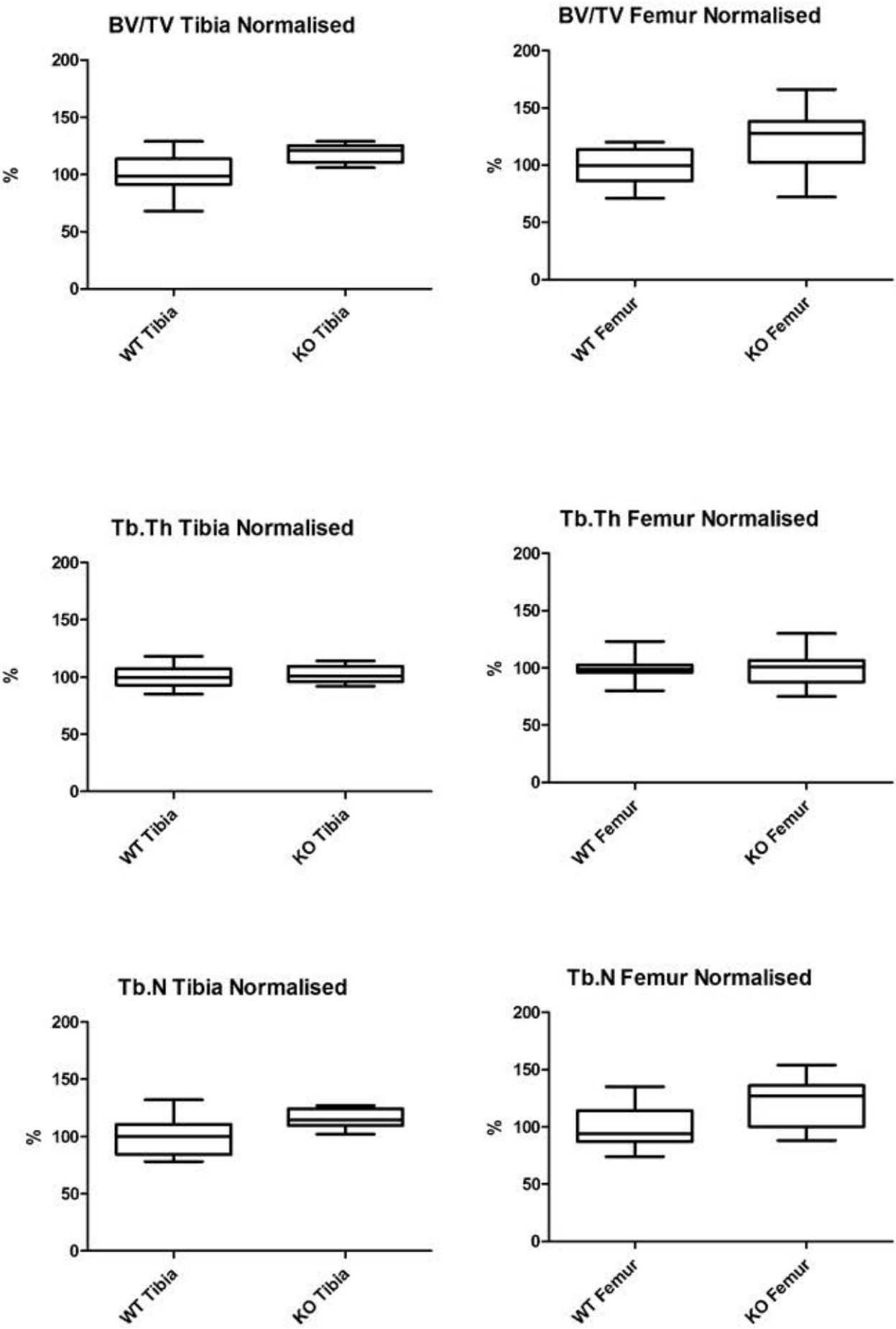

Supplement: Supplementary file 2 — Supplemental Figure 2: Box and whisker plots showing micro CT results. Error bars = +/- SEM. For Tibia measurements, WT n=16 and KO n=12. For Femur measurements WT n=16 and KO n=14. (PDF 65 KB) [file 335_2017_9718_MOESM2_ESM.pdf]
